# Validation of the ADAMO Care Watch for step counting in older adults

**DOI:** 10.1371/journal.pone.0190753

**Published:** 2018-02-09

**Authors:** Daniele Magistro, Paolo Riccardo Brustio, Marco Ivaldi, Dale Winfield Esliger, Massimiliano Zecca, Alberto Rainoldi, Gennaro Boccia

**Affiliations:** 1 School of Sport, Exercise, and Health Sciences, Loughborough University, Loughborough, United Kingdom; 2 National Centre for Sport and Exercise Medicine (NCSEM), Loughborough, United Kingdom; 3 NeuroMuscularFunction | Research Group, School of Exercise and Sport Sciences, Department of Medical Sciences, University of Torino, Torino, Italy; 4 Wolfson School of Mechanical, Electrical and Manufacturing Engineering, Loughborough University, Loughborough, United Kingdom; 5 CeRiSM Research Center ‘Sport, Mountain, and Health’, University of Verona, Verona, Italy; University of Manitoba, CANADA

## Abstract

**Background:**

Accurate measurement devices are required to objectively quantify physical activity. Wearable activity monitors, such as pedometers, may serve as affordable and feasible instruments for measuring physical activity levels in older adults during their normal activities of daily living. Currently few available accelerometer-based steps counting devices have been shown to be accurate at slow walking speeds, therefore there is still lacking appropriate devices tailored for slow speed ambulation, typical of older adults.

This study aimed to assess the validity of step counting using the pedometer function of the ADAMO Care Watch, containing an embedded algorithm for measuring physical activity in older adults.

**Methods:**

Twenty older adults aged ≥ 65 years (mean ± SD, 75±7 years; range, 68–91) and 20 young adults (25±5 years, range 20–40), wore a care watch on each wrist and performed a number of randomly ordered tasks: walking at slow, normal and fast self-paced speeds; a Timed Up and Go test (TUG); a step test and ascending/descending stairs. The criterion measure was the actual number of steps observed, counted with a manual tally counter. Absolute percentage error scores, Intraclass Correlation Coefficients (ICC), and Bland–Altman plots were used to assess validity.

**Results:**

ADAMO Care Watch demonstrated high validity during slow and normal speeds (range 0.5–1.5 m/s) showing an absolute error from 1.3% to 1.9% in the older adult group and from 0.7% to 2.7% in the young adult group. The percentage error for the 30-metre walking tasks increased with faster pace in both young adult (17%) and older adult groups (6%). In the TUG test, there was less error in the steps recorded for older adults (1.3% to 2.2%) than the young adults (6.6% to 7.2%). For the total sample, the ICCs for the ADAMO Care Watch for the 30-metre walking tasks at each speed and for the TUG test were ranged between 0.931 to 0.985.

**Conclusion:**

These findings provide evidence that the ADAMO Care Watch demonstrated highly accurate measurements of the steps count in all activities, particularly walking at normal and slow speeds. Therefore, these data support the inclusion of the ADAMO Care Watch in clinical applications for measuring the number of steps taken by older adults at normal, slow walking speeds.

## Introduction

Walking is one of the most common and important human movements related to living an active and independent life [1,2]. Moreover, it is considered the primary leisure-time physical activity performed by older adults [3,4]. Furthermore, walking is common even among seniors who report no leisure-time physical activity because many activities of daily living require ambulation [5]. However, during the ageing process there is a general functional decline [6,7] and walking performance starts to decline in terms of a decrease in speed, lower cadence, shorter stride length, and an increase time spent in double-limb support [7]. Consequently, locomotion factors, such as rhythm, variability, phases, pace and base of support, may be also affected [8,9]. Given this, walking speed may act as a marker of general wellbeing whereby slow gait speed might lead to a sub-clinical impairment in health status [10,11].

Accurate instruments are required to monitor physical activity levels and evaluate the effects of lifestyle behaviours and specific interventions in older adults [12–15]. Studies of physical activity in older adults have typically assessed behaviours using self-report methods, such as diaries or questionnaires because of their practicality, low cost, low participant burden and wide acceptance [16–19]. However, these approaches have limitations because self-report instruments suffer from significant reporting bias attributable to a combination of social-desirability bias and the cognitive challenge associated with the estimation of frequency and duration of physical activity [17]. In fact, unstructured lifestyle-related activities are often encouraged in public health promotion campaigns [4]; however, these light-intensity activities are more difficult to recognise and less likely to be recalled when self-reporting than structured physical activities [20]. Thus, the accurate assessment of these types of daily activities is important. An alternative approach to a self-reported measure is the objective monitoring of physical activity using wearable devices such as accelerometers and pedometers [21–23]. Wearable activity monitors, such as pedometers, may serve as affordable and feasible instruments for measuring physical activity levels in older adults during physical activity and their daily routine and to nudge users towards a more active lifestyle.

In fact, the use of step counting devices to quantify physical activity has grown over the past decade. However, there are limited data regarding the accuracy of step counting instruments used by older adults. Commercial step counting devices have been shown to be reliable for individuals who walk faster than 0.9 m/s [21,24–26]; however, there are few devices that have been shown to be accurate at walking speeds slower than this [3]. In general, pedometers such as the SC-StepMX (Steps Count, Deep River) and the NL-2000 (New-Lifestyles Inc, Lees Summit, MO, USA) are more accurate at slower walking speeds (0.8 m/s) than accelerometer-based step counting devices [27–29]. Accelerometer-based step counting devices are able of detect both static and dynamic accelerations, thus providing more detailed information than pedometers in the form of time-stamped step counts and activity intensity. In this way they can provide more comprehensive and precise information about mobility patterns, postural changes, and physical activity intensity levels over time. As a result, accelerometer-based step counting devices might be ideal for use with populations who typically engage in very light or brief daily activities, such as the older adults. However, popular accelerometer-based step counting devices such as the ActiGraph GT3X+, the de facto standard instrument to measure physical activities, underestimated the step count by approximately 40% at 0.9 m/s, and by 60% at 0.67 m/s [30]. Also, Paul et al [31] showed that ActiGraph accelerometer undercounted steps in older adults while the Fitbit tracker (One or Zip) was sufficiently accurate (<10% error). However, they did not conduct a specific validation over different walking speeds, and therefore it is not clear whether it could work for lower speeds as well. Despite of the widespread use and popularity of different accelerometer-based step counting devices, there is still lacking for appropriate devices tailored for slow speed ambulation, typical of older adults.

The ADAMO system (Caretek S.r.l., Torino, Italy) is an accelerometer-based monitoring system specifically designed for older adults. It is composed by two elements: a base station, installed in the house of the older adult, and a care watch. The base station receives the data recorded by the ADAMO Care Watch via radio and transmits them to the to the service centre. The ADAMO Care Watch is a device with an embedded algorithm for the measurement of physical activity in older people by counting the steps they take. The step detection algorithm is specifically designed to be sensitive to slow speed ambulation, typical of old age. The number of steps taken is included in the algorithm embedded in ADAMO Care Watch to quantify the mobility index of the individuals. This index is an important marker of the older adult’s general health status. It can be transmitted, through the ADAMO Service Centre, to the family of the older adults or to the health care system to remotely provide information about general health status. The development and validation of the ADAMO Care Watch is part of the SPRINTT (Sarcopenia & physical frailty in older people: multi-component treatment strategies) project (9^th^ Call IMI 2013). Indeed, ADAMO Care Watch acted as the physical activity monitoring system for the living labs developed within the SPRINTT project. Therefore, the aim of this current study was to assess the validity and reliability of ADAMO Care Watch in counting the number of steps taken at different walking speeds and under different mobility conditions. It was hypothesised that there would be no statistically significant difference between ADAMO Care Watch and the actual step count (criterion standard) at slow and normal walking speeds and the mobility tasks in the older adults group. Finally, it was hypothesised that the validity and reliability of the ADAMO Care Watch would decrease at faster walking speeds.

## Materials and methods

### Participants

The participants were recruited through advertisement at Torino University. The participants did not receive incentives (economic or otherwise) for their participation.

The eligibility criteria were: first, being 65 or more years old for the older adult group, and being 20–40 years old for the young adult group; second, all participants were required to walk 100 meters without assistance. Exclusion criteria included a myocardial infarction and/or coronary bypass surgery within the previous year, uncontrolled diabetes or hypertension, orthopaedic impairment or an upper or lower extremity fracture within the past six months, neurological conditions, or current participation in another study. These criteria were intended to ensure that any differences in pedometer validity were primarily due to walking speed and to prevent any potential health and safety issues. Ethical approval for this study was obtained from the local Ethical Review Board of Torino, and all participants were informed that participation in the study was voluntary and confidential. All participants provided their written informed consent in accordance with Italian law.

### Step counting device

The step detection algorithm was embedded within the ADAMO Care Watch. The algorithm exploits the features of a 3-axis accelerometer (ADX346, Analog Devices, Norwood, MA, USA) sampling at 50 Hz. The collected samples were filtered and processed in real-time via a dedicated firmware routine. The processing algorithm was based upon a Dynamic Threshold and Dynamic Precision approach. The raw acceleration signals were continuously processed in bursts of 32 samples. First, a 2-tap finite impulse response filter was applied. The accelerometer is mounted on the watch so that the when the arm is alongside the body, that is the most usual position when walking, the x axis is on the vertical direction, the y axis is on the anterior-posterior direction and z axis is in the medio-lateral (frontal) direction. Then the tri-axial signals were combined, giving a greater weight to the vertical axis. This weighting factor is important because the vertical oscillations of the arm during walking act as a surrogate for the rise and fall of the centre of mass and are deemed to be the most costly in terms of energetics (work). The maximum and minimum values of the bursts were retrieved and the average value, (Max + Min)/2, was estimated (the dynamic threshold level). This threshold level, which was dynamically estimated every 32 samples, was used to decide whether steps had been taken. The occurrence of a step was defined as a negative slope of the combined acceleration pattern when the acceleration curve crossed below the dynamic threshold.

### Measures

#### 30-metre walking tasks

A 30 meters indoor straight track was established in a quiet hallway in the facility. After a single, normal-pace practice trial (not wearing the ADAMO Care Watch), the participants performed the walking tasks wearing the devices at three different self-paced speeds. Specifically, participants were instructed to walk the length of the course: 1) at a normal pace, neither fast nor slow (Normal); 2) slow pace (Slow), i.e. walking slower than normal; 3) fast pace (Fast), i.e. walking faster than normal but without overexertion. To avoid performance bias, the order of the speeds for each participant was randomly chosen using a random number generator. This protocol was designed to test each individual on their own self-determined walking speed range. From a static each task began when the ‘go’ signal was given [32,33], and ended when the participant reached the end area of the 30 meter track. Walking speed (in m/s) was calculated by dividing 30 meters by the elapsed time (in seconds) and acceleration and deceleration phases were included in the calculation of the gait speed [32,33].

#### Timed Up and Go test

The TUG test was conducted to assess mobility, defined as the ability to move independently around the environment (Shumway-Cook *et al* 2005). A 3 meter distance was measured out on the floor in front of a fixed, armless chair, and a cone was placed as a marker at the end of this distance. The ICC of the TUG test has previously been assessed as > 0.95 [34]. The participants were instructed to stand up, walk at a comfortable speed towards the cone, walk around the cone, and walk back and sit down again. The test ended when the participants’ buttocks touched the surface of the seat.

#### Step test

A single step was placed on the floor and participants were instructed to step up and down alternately with their left and right legs 20 times at a self-determined normal stepping rate. The step reflected the standard dimensions of Italian stairs, with a height of 0.18 m and depth of 0.30 m. The inter-rater reliability (ICC) of the step test has been shown to be 0.98 [35].

#### Ascending and descending the stairs

Participants walked up and down stairs (15 steps) at a self-paced normal speed; again, each step was 0.18 m in height and 0.30 m in depth, with a total vertical displacement of 2.70 m. The participants were instructed to step alternately with their left and right leg, and they were not allowed to hang onto a railing when walking up and down the stairs.

### Experimental protocol

Participants wore their own comfortable walking or running shoes. Demographic data were self-reported at the beginning of the study visit. Height was assessed by an anthropometer [36] and weight and BMI using a Tanita Body Composition Analyzer BF-350.

The test procedure took approximately 60 minutes for each participant. All participants performed a 30-metre walking task [37] at three different speeds and a Timed Up and Go (TUG) test [38]. The older adults also performed a stair climbing task [16] and a step test [34,35]. Each task was performed three times, and the tasks were performed in a randomised order. During all tasks, the participants wore two ADAMO Care Watches, one on each wrist, to check for differences due to possible dominant arm asymmetry.

The number of steps detected by the ADAMO Care Watch was recorded at the end of each task, and the device was reset to zero before the start of the successive task. Actual steps taken (i.e., the criterion) were directly observed and recorded using a hand-held tally counter. All tasks were video-recorded for offline verification purposes to ensure the steps were counted in a correct way. To determine walking speed and evaluate the TUG test performance, an experienced technician recorded the duration of each task using a stopwatch, with the help of the recorded video if necessary. Before starting each test, the participants received a detailed briefing and demonstration.

### Statistical analysis

Validity was measured by the level of agreement between the number of steps counted by the pedometer and the criterion measure (direct observation), the actual number of steps as counted by a researcher with a tally counter [3]. The validity of each task’s trials was determined using absolute percentage error scores calculated for each ADAMO Care Watch as follows:

%Error=[(pedometerstepcount−actualstepcount)/(actualstepcount)]×100

A positive value indicated an overestimate in the detection of steps (extra steps detected), and a negative value indicated an underestimate (missed steps). Values close to zero were considered to indicate more accurate results. Mean percentage errors for each task at each speed were calculated by averaging the absolute percentage error across all participants. A repeated measures ANOVA was performed between the three speeds, considering the average time of each trial, across the total sample and in both age groups, in order to confirm that the three self-selected speeds resulted in significantly different times. The Bonferroni correction was applied in post-hoc comparisons used to identify specific group differences. Correlation analysis was performed to investigate a possible relationship between the speed of the walking task and the reliability of the ADAMO Care Watch. Following the identification of a significant relationship, polynomial regression analysis was used to establish the best fit model of the effect of speed on reliability of the care watch. The reliability of ADAMO Care Watch was assessed using intraclass correlation analysis. Two-way mixed intraclass correlation (absolute agreement) coefficients (ICC(3,2)) [39] were calculated for each ADAMO Care Watch over all tasks to demonstrate the consistency of measurements. To assess systematic variation between pedometers and direct observation (manual count) of the different walking speed and TUG, Bland–Altman plots [40] were derived for each ADAMO care watch against the criterion. Data are reported as mean ± SD. Differences between estimates are reported as mean (CI 95%). The level of significance was set as *p*<0.05 in all statistical tests. Statistical Package for Social Sciences (SPSS 20.0 for Windows, IBM, Armonk, NY, USA) was used for all the statistical analyses.

## Results

A total of 40 people participated in the study: 20 older adults (10 females) ages 65 years or older (mean ± SD, 75 ± 7 years; range, 68–91) and 20 young adults (10 females, 25 ± 5 years, range 20–40) were recruited for this study. The mean body mass index, in line with Italian normative data [41], was 27.4 ± 5.4 kg m^-2^ for the older adult group and 22.4 ± 3.2 kg m^-2^ for the young adult group. The times and speeds for the walking task trials and TUG tests are presented in Table 1. The speed data are reported with two units (m/min and m/s) to facilitate comparison with previous studies [3,20,25,42].

**Table 1:** 30-meters walking tasks (Normal, Slow, Fast) and TUG performances in times and speeds.

	Task	Time (s)		Speed (m/min)		Speed (m/s)	
		*M*	*SD*	*M*	*SD*	*M*	*SD*
Total sample (N = 40)	Walk Normal	24.80	4.65	74.89	12.94	1.24	0.21
	Walk Slow	33.57	6.95	55.59	10.04	0.92	0.16
	Walk Fast	18.62	4.76	102.02	22.07	1.70	0.36
	TUG test	9.61	1.90				
Older adult group (N = 20)	Walk Normal	27.68	4.61	66.71	10.93	1.11	0.18
	Walk Slow	37.94	7.19	49.06	9.21	0.81	0.15
	Walk Fast	21.90	4.56	85.38	16.37	1.42	0.27
	TUG test	10.77	1.89				
Young adult group (N = 20)	Walk Normal	21.91	2.36	83.07	9.14	1.38	0.15
	Walk Slow	29.20	2.69	62.13	5.69	1.03	0.09
	Walk Fast	15.34	1.79	118.67	12.20	1.97	0.20
	TUG test	8.45	1				

Data presented as Means (M) and Standard Deviation (SD); speed is presented in metres/minute (m/min) and metres/second (m/s); The time is in seconds (s).

The repeated measures ANOVA showed a significant difference between the three walking speeds (Normal, Slow, Fast) in the whole sample (*F* = 224.205, *p*<0.0001, η^2^ = .922) and in both the older adult (*F* = 78.424, *p*<0.0001, η^2^ = 0.897) and young adult group (*F* = 246.08, *p*<0.0001, η^2^ = 0.965). A post-hoc analysis, with Bonferroni adjustment, revealed significant differences in the whole sample and in both groups separately in Normal versus Slow speed, Normal versus Fast speed and Slow versus Fast speed (all *p*<0.0001).

Table 2 presents the percentage error scores calculated for the ADAMO Care Watch worn on the right (R) and left (L) arms for each task. The mean percentage error for the 30-metre walking tasks was greater at faster speeds in both the young adult group and the older adult group In the TUG test, the older adult group showed a smaller percentage error than the young adult group. For more details see Table 2.

**Table 2:** Percent error scores calculated for each side and each task.

	Task	Actual steps taken		Steps Right pedometer		Steps Left pedometer		Error Right pedometer (%)		Error Left pedometer (%)	
		*M*	*SD*	*M*	*SD*	*M*	*SD*	*M*	*SD*	*M*	*SD*
Total sample (N = 40)	Walk normal	45	7	44	7	44	7	-2.12	2.41	-1.97	2.84
	Walk slow	52	7	51	7	51	6	-1.55	5.05	-1.10	2.30
	Walk fast	39	8	33	12	33	12	-17.70	20.77	-16.77	20.62
	TUG test	12	2	12	3	12	3	-3.96	7.70	-4.73	8.65
Older adult group (N = 20)	Walk normal	50	6	49	6	49	6	-1.86	2.70	-1.28	2.24
	Walk slow	57	5	56	5	56	5	-1.34	2.36	-1.51	2.45
	Walk fast	45	7	42	7	42	7	-5.90	5.99	-7.43	8.23
	TUG test	14	2	13	2	13	2	-1.28	7.31	-2.23	7.62
	Step Test	20	-	18	3	19	3	-8.07	13.20	-4.47	13.62
	Stairs go up	16	-	17	2	15	2	16.85	1.97	14.82	2.18
	Stairs go down	16	-	14	4	13	4	14.12	3.8	13.13	4.12
Young adult group (N = 20)	Walk normal	40	3	39	3	39	3	-2.38	2.14	-2.66	3.25
	Walk slow	47	3	46	5	47	3	-1.76	6.8	-.69	2.11
	Walk fast	33	3	23	8	24	9	29.50	23.61	26.11	24.94
	TUG test	11	1	10	2	10	2	-6.64	7.30	-7.24	9.08

*Notes*: Data presented as Means (M) and Standard Deviation (SD).

"—" represents the absence of SD due to the fixed number of steps during ascending and descending task

The ICCs calculated for each ADAMO Care Watch and each task trial are presented in Table 3. For the whole sample, the ICCs for the ADAMO Care Watch for the 30-metre walking tasks at each speed and for the TUG test showed a range between 0.931 to 0.985 (for more details see Table 3).

**Table 3:** ICC scores calculated for each ADAMO Care Watch across tasks.

	Task	Consistency					
		Right arm care watch			Left arm care watch		
		ICC	95% CI		ICC	95% CI	
			Lower Bound	Upper Bound		Lower Bound	Upper Bound
Total sample (N 40)	Walk normal	0.981	0.96	0.99	0.979	0.96	0.98
	Walk slow	0.972	0.95	0.98	0.962	0.93	0.97
	Walk fast	0.976	0.95	0.98	0.985	0.97	0.99
	TUG test	0.931	0.88	0.96	0.945	0.91	0.97
Older adult group (N 20)	Walk normal	0.980	0.96	0.99	0.975	0.95	0.99
	Walk slow	0.960	0.92	0.98	0.938	0.87	0.97
	Walk fast	0.949	0.89	0.98	0.961	0.92	0.98
	TUG test	0.914	0.82	0.96	0.927	0.85	0.97
	Step Test	0.848	0.68	0.94	0.850	0.69	0.94
	Stairs go up	0.844	0.66	0.94	0.863	0.71	0.94
	Stairs go down	0.974	0.95	0.99	0.968	0.93	0.99
Young adult group (N 20)	Walk normal	0.918	0.76	0.97	0.879	0.75	0.95
	Walk slow	0.918	0.83	0.97	0.899	0.79	0.96
	Walk fast	0.934	0.86	0.97	0.970	0.94	0.99
	TUG test	0.828	0.64	0.93	0.873	0.74	0.95

*Notes*: Data presented as intraclass correlation coefficient (ICC) and confidence interval (CI).

**Fig 1:**
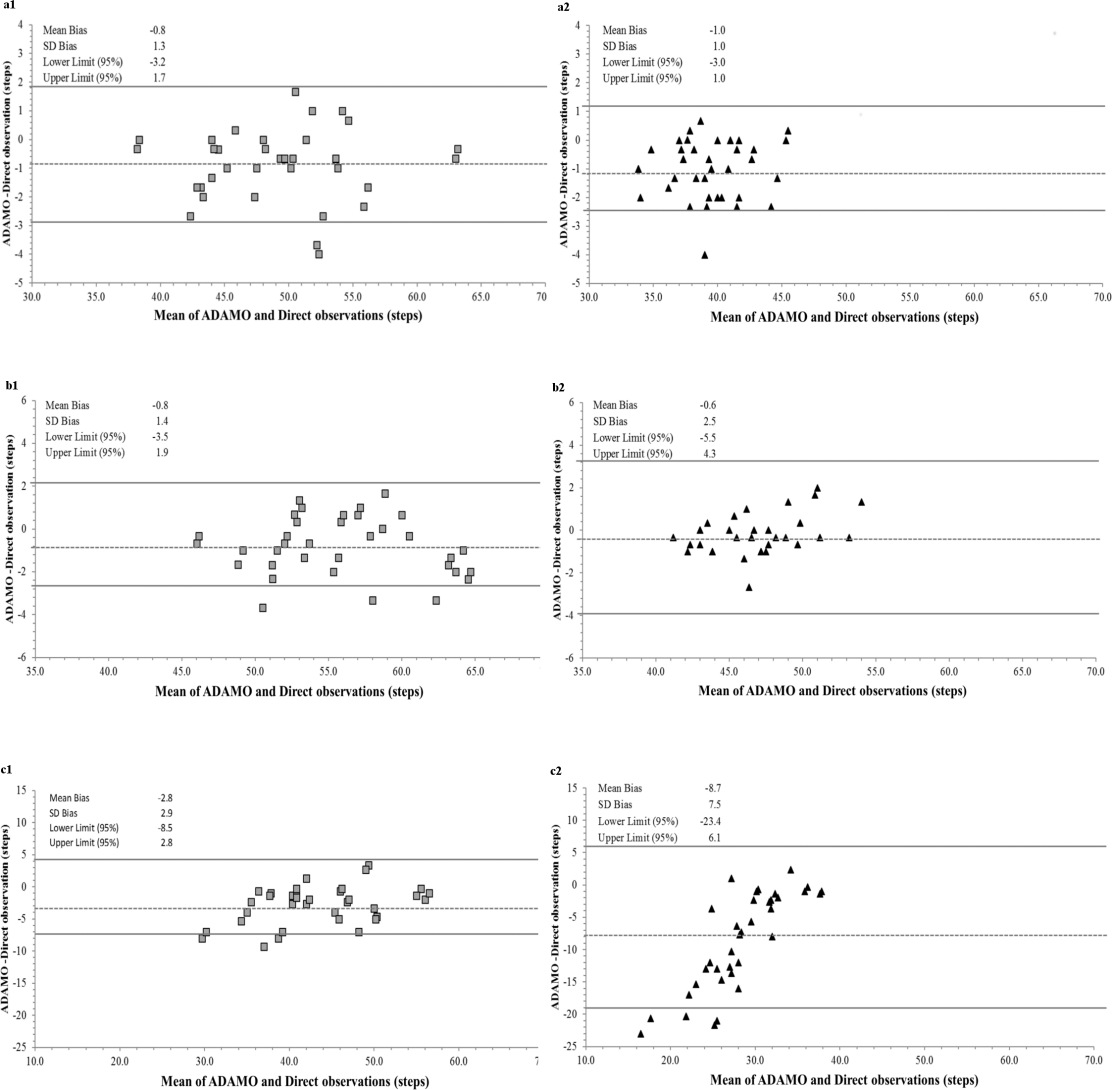
Bland–Altman plots between criterion and ADAMO Care Watch during walk test. a) Normal speed (*a1* Older adults and *a2* Young adults); b) Slow speed (*b1* Older adults and *b2* Young adults); c) Fast speed (*c1* Older adults and *c2* Young adults). Dashed lines represent mean bias differences; Solid lines represent 95% prediction intervals.

**Fig 2:**
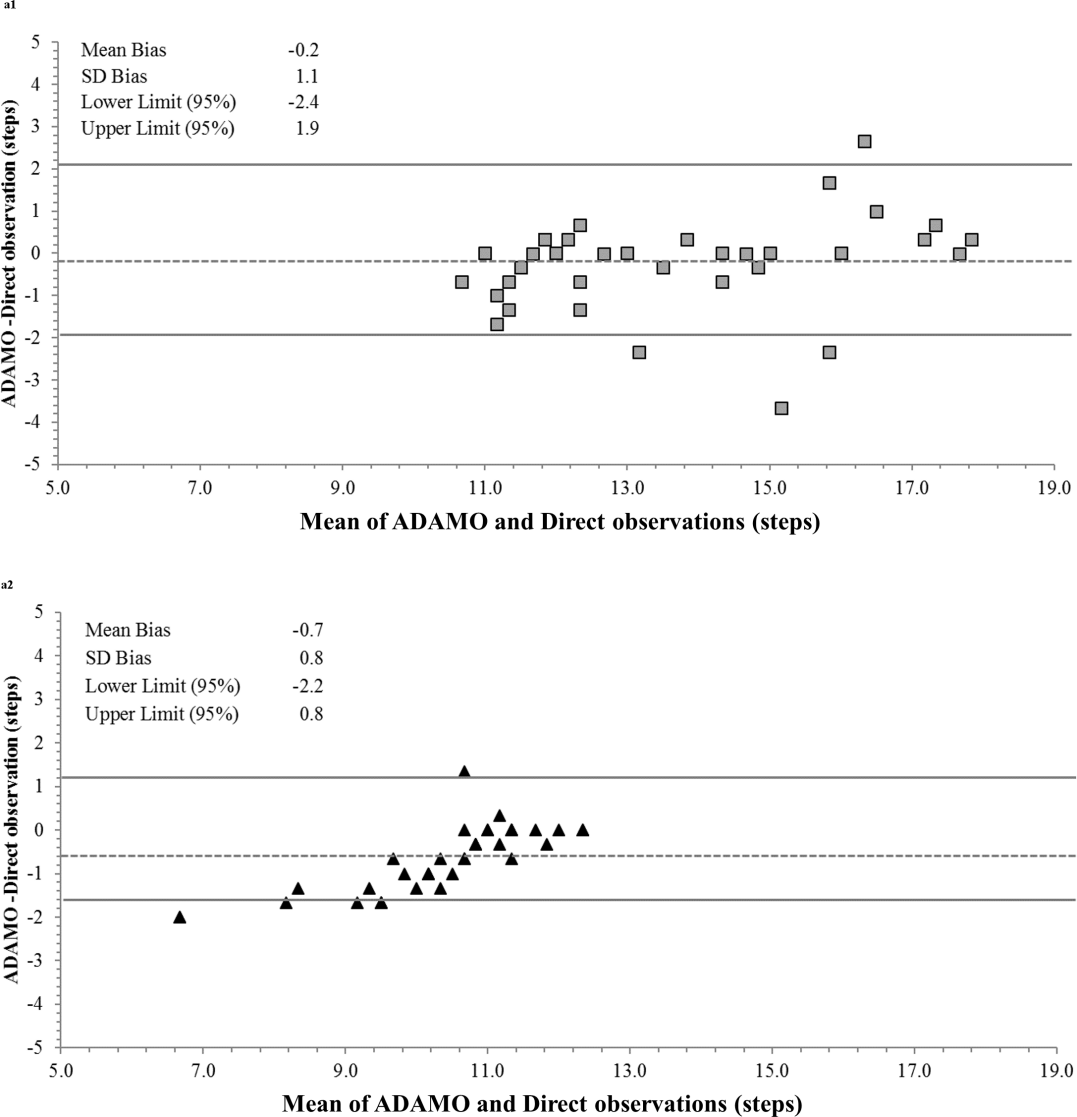
Bland–Altman plots between criterion and ADAMO Care Watch during TUG test. Dashed lines represent mean bias differences; Solid lines represent 95% prediction intervals; *a1* represent Older adults and *a2* represent Young adults.

Bland–Altman plots were constructed to assess the agreement between ADAMO Care Watch against the criterion during walking at normal, slow, and fast speeds (Fig 1) and TUG (Fig 2). Limits of agreement were narrow across normal and slow walking pace and TUG in both groups. Wide limits of agreement with larger scatter were observed during the walking at fast speeds, especially in the young adult group where the absolute speeds were higher.

**Fig 3:**
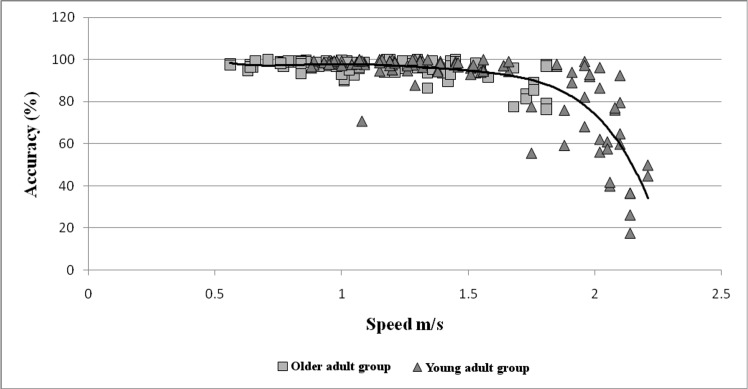
Regression model: Association of speed on reliability of ADAMO Care Watch of the whole sample.

Correlation analysis showed a significant negative relationship between the speed of the walking task and the reliability of the ADAMO Care Watch (*r* = −0.647, *p*<0.0001). Polynomial regression analysis showed the best model that could represent the association of the speed on the reliability of the ADAMO Care Watch (*r*^*2*^ = −0.667, *p*<0.0001, *F* = 149.70) (Fig 3) and the relationship was clearly nonlinear.

## Discussion

The present study examined the validity of the ADAMO Care Watch in measuring steps taken during a 30-metre walking task at different speeds and during various mobility tasks (a TUG test, step test and stair climbing) in groups of older and young adults. The main finding was that the ADAMO Care Watch demonstrated highly accurate measurement of actual steps taken during the walking tasks at slow and normal speeds (range 0.5–1.5 m/s), showing an absolute error from 1.3% to 1.9% in the older adult group and from 0.7% to 2.7% in the young adult group. The results of the walking task at fast speed (1.5–2.3 m/s) showed an absolute error of 6% in the older adults group and 17% in the young adult group.

Although a direct comparison cannot be made between our findings and previously reported data, it is possible to highlight some differences. Given the ADAMO Care Watch was designed for older adults walking at typically slower speeds, only normal and slow speed results in the present study were compared with previous studies that investigated the same speed range. Some previous studies [3,25,43] reported that pedometers showed a percentage error greater than 20% at walking speeds ≤0.9 m/s; in contrast, in the present study the ADAMO Care Watch showed high validity in step detection at slow walking speeds (absolute error of 1.5% at 0.8 m/s), similar to the activPAL accelerometer-based monitor [16,42], which showed a mean percentage error <1% for three speeds in the range 0.7 to 1.6 m/s in older adults. The ADAMO Care Watch showed greater validity than all three devices examined in the study of Storti et al. [20] (mean absolute errors: Yamax, 16.9%; Actigraph, 8.7%; and StepWatch, 5.7%) and speeds (<0.8 m/s, 0.8–1.0 m/s and >1.0 m/s). A similar trend was found by Cyarto et al. [25], who reported mean percentage errors of 25% at 0.95 m/s, 46% at 0.8 m/s, 55% at 0.64 m/s and 74% at 0.4 m/s for the Yamax SW 200 during walking. Recently, Martin et al. [3] reported the performance of five pedometers (Omron HJ 105, Yamax Digiwalker 200, SportLine 330, New-Lifestyles 2000 and the ActiCal Accelerometer) in counting steps at four speeds (0.46, 0.66, 0.85 and 1.31 m/s): the mean percentage errors across all devices were found to be higher (56%, 40%, 19% and 9%, respectively) than those of the ADAMO Care Watch in the present study. Ichnoseki-Sekine et al. [44] showed that the Omron HJ 720-IT pedometer underestimated steps by an average of 53.2% over a range of slow walking speeds (0.28–0.99 m/s) in older adults. Recently, Lee et al. [45] investigated four pedometers using a treadmill at various speeds. Only the Omron HJ-720 T pedometer showed average percentage errors lower than 1.1% at all speeds, with the Actigraph GT3X, Yamax Digiwalker SW-701 and Polar Active accelerometer showing percentage errors greater than 10% at the lowest speeds (0.9 m/s and 1.1 m/s). Moreover, ADAMO Care Watch was shown to be accurate when compared to well-known consumer-based step counters such as the SC-StepMX, the NL-2000 and the Fitbit tracker that demonstrated a good step count accuracy for older adults [27–29,31,46]. The high values of ICCs confirmed the consistency of ADAMO Care Watch measurements, highlighting that the differences observed in the test were mainly due to differences among the participants. Comparing the results for normal and slow speeds with the studies described above that investigated a similar range of speeds, we can conclude that ADAMO Care Watch showed a high level of validity at normal and slow speeds. Moreover, considering that our protocol involved the participants self-selecting their pace at the different speeds, it can be assumed that the speeds chosen were those that are the most common in everyday life. The findings of the present study therefore show that ADAMO Care Watch was accurate in detecting steps during normal walking on a short straight course and during the TUG test.

Furthermore, the results of the present study were deemed to be supportive of the stated hypothesis that fast walking speed reduces pedometer reliability. The regression analysis confirmed that a fast walking speed negatively affected the reliability. ADAMO was designed to be sensitive to low speed, indeed during fast speed ADAMO Care Watch Care Watch seems to significantly underestimate the number of steps taken when compared with a criterion method.

In the TUG test, the ADAMO Care Watch showed a high level of validity and reliability in both the older adult and young adult group. Specifically, the ADAMO Care Watch showed a lower percentage error for the older rather than for the young adult group, corresponding with the slower walking speed.

The TUG test reflects important daily physical functions, such as the ability to rise from a chair and walk around the home and has been shown to predict the development of disability related to mobility functions in older people [47]. The relative complexity of the motor sequence involved in the TUG test acts as a representative indicator/marker of actual daily situations [48].

During the step test and the climbing up and down stairs tasks, the ADAMO Care Watch overestimated the actual number of steps, with a slightly higher percentage error (12.4% to 16.6%). This slight inaccuracy could be attributed to the different spatiotemporal parameters of climbing up and down the stairs compared with walking on a level surface (such as a slower velocity, shorter step length and longer step time). In fact, negotiating stairs during the climbing up and down stairs is a complex locomotor task that imposes significant challenges for movement control in older adults [49]. Compared to level walking, stair ambulation demands greater balance control [50], especially in older adults when motor functions are reduced by the ageing process. Again, the ADAMO Care Watch showed high reliability in these tasks (ICC range 0.81–0.96). The decision to include these tasks in the assessment of the validity of ADAMO Care Watch was due to the fact that ascending and descending stairs are daily activities that can take place in many types of natural and built environments.

There were limitations to the present study. First, we studied the validity of the ADAMO Care Watch in a controlled laboratory environment. Further studies should examine how counting steps in a normal-life situation compares in terms of validity and reliability. Second, the participants were healthy and so the results cannot be generalised to specific populations such as older adults living in long-term care facilities, older adults with mild cognitive impairment, or those with diseases which can affect gait. Third, the walking course was relatively short (30m). Further studies should examine the validity and reliability of ADAMO Care Watch in longer walking paths/trails to extend these results.

There are several strengths of this study. First, to our knowledge, ADAMO Care Watch is the first device specifically marketed for use in the older adult population, with a step detection algorithm specifically designed to be sensitive to slow walking speeds. Further, the accordance of our results with previous studies [16,51] emphasize the fact that the ADAMO Care Watch’s validity and reliability specifically tuned for use in slower walking adults. Second, the high reliability values of the ADAMO Care Watch acts to minimize measurement error. Thirdly, these findings are in line with previous study from Tudor-Locke and Rowe [52] and Floegel and colleagues [46] who underline the need for appropriate device for individual monitoring in specific groups.

## Conclusion

The findings of this study showed that the ADAMO Care Watch demonstrated a valid measurement of the number of steps performed by older adults during mobility tasks such as walking at different speeds and the TUG test and during ascending and descending stairs. Moreover, these findings support the use of the ADAMO Care Watch for measuring the number of steps taken per day by older adults with slower walking speed. In summary, step counting devices are valuable tools and an accessible way to quantify physical activity in a research setting. The popularity of step counting devices as an objective measure of physical activity originates from their ability to provide direct, objective, and physical activity information. Our findings indicate that the ADAMO Care is both a reliable and valid step counting device.

## Supporting information

S1 File Dataset. (XLSX)
